# User Satisfaction with Orthotic Devices and Service in Taiwan

**DOI:** 10.1371/journal.pone.0110661

**Published:** 2014-10-22

**Authors:** Chiung-Ling Chen, Ya-Ling Teng, Shu-Zon Lou, Chung-Hui Lin, Fen-Fen Chen, Kwok-Tak Yeung

**Affiliations:** 1 School of Occupational Therapy, Chung Shan Medical University, Taichung, Taiwan; 2 Occupational Therapy Room, Chung Shan Medical University Hospital, Taichung, Taiwan; University of Perugia, Italy

## Abstract

User satisfaction is afforded considerable importance as an outcome measurement in evidence-based healthcare and the client-centered approach. Several studies have investigated user satisfaction with orthoses. Few studies have investigated user satisfaction with orthoses in Taiwan. Therefore, the purpose of this study was to investigate the user satisfaction with orthotic devices and service using the Taiwanese version of Quebec User Evaluation of Satisfaction with Assistive Technology. We conducted a cross-sectional study of 280 subjects who had used orthoses and received services. The results showed that the mean satisfaction score was 3.74 for the devices and 3.56 for service. Concerning the participants, 69.1% and 59.6% were quite satisfied or very satisfied with their devices and service, respectively. The satisfaction score of orthotic service was lower than that of the devices. Regarding demographic characteristics, participants living in different areas differed only in service score (p = 0.002). The participants living in eastern area and offshore islands were the least satisfied with the orthotic service. For clinical characteristics, there was a significant difference in satisfaction scores among severity of disability (all p = 0.015), types of orthoses (all p = 0.001), and duration of usage (all p = 0.001). The participants with mild disability, wearing the pressure garment and using the orthosis for less than one year, were the most satisfied with their orthotic devices and service. There is a need for improved orthotic devices and services, especially with respect to the comfort of the devices and the provision of subsidy funding.

## Introduction

An orthosis is defined as “an externally applied device used to modify the structural and functional characteristics of the neuromuscular and skeletal systems” [1]. The terms orthosis and splint are often used synonymously in the healthcare field. However, orthosis is a more inclusive term compared to splint, in that it provides a force system for dynamic control in addition to stabilization of the body [2]. In addition, orthosis is a permanent device to substitute for the loss of muscle function, whereas a splint is commonly prescribed for temporary use as part of a treatment program [3]. There are several types of orthoses, and they can be classified according to anatomic site, material, purpose of application, or power source.

Orthoses can be used for patients with neuromuscular and musculoskeletal impairments that result in functional limitation and disability. Overall 35 million Americans have disabling conditions that interfere with their life activities, and 16% of those have an orthopedic impairment. Approximately 20% of individuals with complete or partial paralysis of the extremities use orthoses. Of those with paralysis, 27% are 45–64 years of age. The proportion of the population with some paralysis will increase significantly as the Baby Boomer generation ages [reaching ages 46–64 after 2010] [4]. The total number of people with paralysis, deformities, or orthopedic impairments who use orthoses is expected to reach 7.3 million by 2020 [5]. In Taiwan, the number of people with physical and mental disabilities reached 1.11 million in 2012. Many of these individuals need orthoses to help them cope with limitations that interfere with their ability to complete the activities of daily living. In 2005, the Ministry of the Interior enacted the Act entitled “Regulations on Subsidization for Medical Treatment and Auxiliary Appliances for the Disabled” pursuant to the law entitled “Physically and Mentally Disabled Citizens Protection Act.” These regulations provide subsidization for medical rehabilitation that is not covered by the national health insurance and for auxiliary appliances used to help the disabled to get over the physiological malfunction and promote their self-servicing abilities. These regulations covered orthoses as rehabilitation devices. They included lower limb orthoses, upper limb orthoses, spinal braces, pressure garments, and others. The effectiveness of devices and user satisfaction are important indicators that help the government or policy makers make decisions.

Furthermore, user satisfaction is considered an important outcome measurement in evidence-based healthcare and the client-centered approach [6]–[8]. DeRuyter [9] asserted that the five main outcomes in the field of assistive technology are user satisfaction, clinical results, functional status, quality of life, and costs. User satisfaction can be defined as an attitude about a service, a product, a service provider, or a person's health status [6]. The Quebec User Evaluation of Satisfaction with Assistive Technology (QUEST 2.0) was designed as an outcome measurement instrument to measure user satisfaction with a wide range of assistive technology. This 12-item measure assesses user satisfaction with two components, i.e., Device and Services [10], with items measured on a 5-point satisfaction scale, which has proved to be a reliable and valid instrument for measuring outcomes in the field of assistive technology [11]. The original Canadian English and French versions of QUEST 2.0 were translated into Dutch, Swedish, Norwegian, Danish, Japanese, and Cantonese [12]–[15]. Mao et al. [16] developed and validated the Taiwanese version of QUEST 2.0 (T-QUEST). They identified culture-specific items and added them to QUEST 2.0. The item ‘Cost’, identified as the expense associated with purchasing, maintaining, and repairing the device, was the only culture-specific item incorporated into the service component of T-QUEST. Therefore, T-QUEST includes 13 items, i.e., eight items in the device domain and five items in the service domain. The results of Mao et al.'s research indicated that T-QUEST is a valid and reliable instrument for measuring user satisfaction with various types of assistive technology among Mandarin-speaking individuals in Taiwan.

Several studies have examined user satisfaction with orthoses. One study conducted by Greetzen et al. [17] assessed consumer/patient satisfaction with the services of the prosthetics and orthotics facilities in the northern part of The Netherlands. Other studies examined the users' satisfaction with or opinions about specific orthoses. Those studies included parental satisfaction with the CranioCap [18], consumer opinions of a stance-control knee orthosis, and a hinged ankle-foot orthosis [19,20]. They also included patient satisfaction with lower limb orthoses and custom-molded foot orthoses [21,22]. Some of the studies addressed user satisfaction with hand orthoses for radial nerve injury and rheumatoid arthritis [23,24] and a spinal brace for scoliosis [25]. Few studies have been conducted to investigate user satisfaction with orthoses in Taiwan. The research data were scarce since most of the research related to user satisfaction focused on daily living assistive devices but not the orthotic devices and the research evidence were based on anecdotal evidence from personal interview or online surveys. Therefore, the purpose of this study was to investigate the user satisfaction with orthotic devices and service using the T-QUEST. The orthotic devices included lower limb orthoses, spinal braces, and pressure garments. These devices are auxiliary appliances that are funded through the government welfare allowance, and they are prescribed commonly for non-temporary problems of persons with disabilities. In contrast to the lower-limb orthoses that address walking and running issues, the upper limb orthoses are involved in a greater variety of activities. The types of upper limb orthoses vary because of the complexity of the functions provided by the hands and upper limbs. In addition, very few disabled people use upper limb orthoses that are covered in the benefits regulations. Therefore, user satisfaction with upper limb orthoses was not investigated in this study.

In summary, user satisfaction is an important outcome in evidence-based and client-centered practice. In addition, different subsidy policies or regulations in different countries may influence user satisfaction. Therefore, the purpose of this survey was to describe user satisfaction with orthosis and service offered to subjects with disability who are receiving orthosis and service subsidized by the government in Taiwan. Further objectives were to determine whether demographic (age, gender, area, education level) and clinical (severity, type of orthosis, duration of usage) characteristics influence satisfaction. By investigating users' satisfaction and collecting users' opinions, we hope to influence the policy makers to establish policy and funding regulations.

## Methods

### Ethics statement

This study has been reviewed by the Chung Shan Medical University Hospital Institutional Review Board. A signed informed consent form was obtained from all participants after clearly introducing the survey. For those aged below 20, a consent form was also obtained from their parents.

### Sample

The sample was selected using convenience sampling. It included those who were receiving services at assistive technology resource centers or prosthetics and orthotics centers. The inclusion criteria were that participants had disability card (i.e., with non-temporary disability), received an orthosis subsidized by the government, and used orthosis for at least one month. In order to include sample characteristics that might vary by region, participants were recruited from centers that were geographically dispersed across Taiwan, including the northern, central, southern, and eastern districts (including offshore islands). We expected to collect total of 300 samples. The number of samples in each area was determined based on the geographical distribution of persons with physical disabilities. The clinical staffs at four centers in each area were invited to help in collecting data.

### Participants' characteristics

A demographic/clinical form was designed to collect information. The demographic information included age, gender, area of Taiwan, highest education level attained (with response categories being non-high school graduate, high school graduate, and college graduate), living arrangement (with response categories being alone, with family, institute, and miscellaneous), and work status (with response categories being competitive, supported, sheltered, unemployment, and miscellaneous). The age was also coded in five groups (below 30, 30–39, 40–49, 50–59, 60 and above). The clinical information included orthosis-related diagnosis, severity of disability (with response categories being mild, moderate, and severe), type of orthosis, duration of usage (with response categories being below 1y, 1–3y, 3–5y, 5y and above), and frequency of usage. The severity of disability was recorded from the record in the disability card. The degree of severity in the disability card was identified by the physician according to the disability eligibility system. Both profundus and severe degrees were recorded as severe category. If the participant had more than one orthosis, she/he was asked to information about one of them.

### Types of orthoses

The types of orthoses investigated in this study included lower limb orthoses, spinal braces, and pressure garments. The categories of lower-limb orthoses included foot orthoses, ankle-foot orthoses (either high-temperature thermoplastic or metal), knee-ankle-foot orthoses, and hip-knee-ankle-foot orthoses.

### Instrument

T-QUEST, which provides a total score and subscores for the device and for the service [16], was used to investigate user satisfaction. It is generic and can be self-administered or administered in an interview situation. T-QUEST comprises eight items measuring satisfaction with the devices (dimensions, weight, adjustments, safety, durability, ease of use, comfort, and effectiveness) and five items measuring satisfaction with service (service delivery, repairs and servicing, professional services, follow-up, and cost). Each item is scored on a 5-point ordinal satisfaction scale, i.e., 1 =  not satisfied at all; 2 =  not very satisfied; 3 =  more or less satisfied; 4 =  quite satisfied; and 5 =  very satisfied. Space for comments is provided next to each item. The participants were asked to explain any dissatisfaction that had a score of less than 4. In the final section of the questionnaire, the participants were asked to select three satisfaction items that are considered the most important for the device being assessed. The participants were also invited to provide opinions or additional suggestions for the use and service of orthoses.

### Data collection and analysis

The demographic/clinical form and the T-QUEST were completed via self- or interviewer-administration. The choice of administration format depended on the characteristics of the participants (if they were able to read and write), and it was determined by the clinical staff of the centers. The clinical staff conducted interviews or facilitated the administration process, answer any question, and check for missing data when the questionnaires were self-administered. The staff had to make sure that participants completed all questions (i.e., none was left blank) by discussing the missing items with the participants and encouraging them to provide a response.

After the participants completed the questionnaires and returned them to the researchers, the answers to the items were entered in a database, and the data were processed anonymously. If more than 6 satisfaction items unanswered, the questionnaire was considered invalid. The score of missing items in the valid questionnaires was replaced by the mode of the scores for non-missing items. T-QUEST scores were analyzed both as subscales for the device and for the service and as an item-by-item analysis. Descriptive statistics and non-parametric statistics were used. Descriptive statistics, such as frequencies, means, and standard deviations, were computed for the characteristics and satisfaction scores reported by the participants. The percent frequency of each of the three most important items was also calculated. The Wilcoxon Signed Ranks Test was used to compare the mean satisfaction scores for device and service domains. The Mann-Whitney U-test (when assessed in two categories) and the Kruskal-Wallis Test (more than two categories) were used to compare the differences in satisfaction scores between the subgroups of gender, age, area, education level, severity of disability, type of devices and duration of usage. The Mann-Whitney U-test was also used as a post-hoc test for pair-wise comparisons when the Kruskal-Wallis test shows a significant difference between the groups. All statistical analyses were performed using SPSS 14.0 for Windows. A p-value of 0.05 was considered significant.

## Results

### Participants' characteristics

Two hundred and eighty valid questionnaires completed by participants (160 males and 120 females) were included in the analysis. The mean age of participants was 44.7 (SD = 13.0, range 8–90) and 57.1% were males. Most of the participants were polio survivors (47.1%). Various diagnoses, such as lumbar spondylosis, scoliosis, and sciatica were included in the miscellaneous group. Eighty-two percent of the participants wore their orthoses every day. Regardless of whether the participants obtained their orthoses from the assistive technology or orthotics centers, almost all participants received orthotic services, such as prescription, assessment, measurement, or fitting of orthosis during application. The demographic and clinical characteristics of the participants were presented in Table 1.

**Table 1:** Demographic and clinical characteristics of the participants.

Demographic characteristics	number	percent
Total	280	100
Gender		
Male	160	57.1
Female	120	42.9
Age (years)		
<30	39	13.9
30–39	44	15.7
40–49	97	34.6
50–59	69	24.6
≥60	31	11.1
Area of Taiwan		
Northern	101	36.1
Central	71	25.4
Southern	88	31.4
Eastern and Offshore islands	20	7.1
Level of education		
Non-high school graduate	82	29.3
High-school graduate	109	38.9
College graduate	89	31.8
Living arrangement		
Alone	23	8.2
With family	240	85.7
Institution	12	4.3
Miscellaneous	5	1.8
Work status		
Competitive employment	138	49.3
Supported employment	3	1.1
Sheltered employment	10	3.6
Unemployment	126	45.0
Miscellaneous	3	1.1
Diagnosis		
Spinal cord injury	37	13.2
Stroke/brain injury	32	11.4
Cerebral palsy	8	2.9
Polio	132	47.1
Lower limb orthopedic disorders	13	4.6
Burn	34	12.1
Miscellaneous	24	8.6
Severity of disability		
Mild	73	26.1
Moderate	112	40.0
Severe	95	33.9
Types of orthoses		
Foot orthosis (FO)	30	10.7
Ankle-foot orthosis (AFO)	57	20.3
Knee-ankle-foot orthosis (KNAFO)	85	30.4
Hip- knee-ankle-foot orthosis (HKAFO)	36	12.9
Spinal brace (SB)	38	13.6
Pressure garment (PG)	34	12.1
Duration of usage		
<1y	110	39.3
1–3y	69	24.6
3–5y	51	18.2
≥5y	50	17.9
Frequency of usage (days/week)		
<1	24	8.6
1–6	26	9.3
7	230	82.1

### User satisfaction

Table 2 shows the ratings of the participants on all 13 items divided into the two domains. Overall, the most participants indicated that they were satisfied with their devices and service. The percentages of participants who indicated ‘quite satisfied’ (4 on the scale of 1 to 5) or ‘very satisfied’ (5 on the scale of 1 to 5) were combined to determine a percentage of individuals who are satisfied. There were 69.1%, 59.6%, and 65.3% of participants satisfied with their device, service, and total respectively. Only a few participants were not satisfied at all with device (1.6%), service (2.8%), and total (2.0%).

**Table 2:** Number and percentage of participants who rating the T-QUEST.

	1	2	3	4	5
	_n (%)_	_n (%)_	_n (%)_	_n (%)_	_n (%)_
Device (mean)	4.4 (1.6)	20.6 (7.4)	61.6 (22.0)	150.5 (53.8)	42.9 (15.3)
Dimension	1 (0.4)	15 (5.4)	63 (22.5)	163 (58.2)	38 (13.6)
Weight	7 (2.5)	27 (9.6)	69 (24.6)	140 (50.0)	37 (13.2)
Adjustment	6 (2.1)	21 (7.5)	53 (18.9)	162 (57.9)	38 (13.6)
Safety	0 (0.0)	13 (4.6)	46 (16.4)	166 (59.3)	55 (19.6)
Durability	1 (0.4)	16 (5.7)	67 (23.9)	148 (52.9)	48 (17.1)
Ease of use	5 (1.8)	22 (7.9)	58 (20.7)	150 (53.6)	45 (16.1)
Comfort	13 (4.6)	33 (11.8)	81 (28.9)	119 (42.5)	34 (12.1)
Effectiveness	2 (0.7)	18 (6.4)	56 (20.0)	156 (55.7)	48 (17.1)
Service (mean)	7.8 (2.8)	35.6 (12.7)	69.8 (24.9)	125.6 (44.9)	41.2 (14.7)
Service delivery	5 (1.8)	23 (8.2)	72 (25.7)	140 (50.0)	40 (14.3)
Repairs & services	2 (0.7)	28 (10.0)	63 (22.5)	138 (49.3)	49 (17.5)
Professional services	2 (0.7)	19 (6.8)	60 (21.4)	142 (50.7)	57 (20.4)
Follow-up	9 (3.2)	38 (13.6)	81 (28.9)	117 (41.8)	35 (12.5)
Cost	21 (7.5)	70 (25.0)	73 (26.1)	91 (32.5)	25 (8.9)
Total (mean)	5.7 (2.0)	26.4 (9.4)	64.8 (23.1)	140.9 (50.3)	42.2 (15.1)

Note. n: number of participants, Satisfaction scores range from 1 to 5 with 1 ‘not satisfied at all’, 2 ‘not very satisfied’, 3 ‘more or less satisfied’, 4 ‘quite satisfied’ and 5 ‘very satisfied’.

Table 3 shows the means and standard deviations of satisfaction scores for each of the items of T-QUEST for each orthosis separately and for all orthoses combined. User satisfaction with the orthotic device and with the service was medium to high. Mean scores for users' satisfaction were 3.74 (SD = 0.64) for their devices, 3.56 (SD = 0.76) for service, and 3.67 (SD = 0.64) for total. The mean scores for each item ranged from 3.10 (‘Cost’) to 3.94 (‘Safety’). The participants were satisfied most with ‘Safety’, followed by ‘Professional service’ and ‘Effectiveness.’ They expressed the least satisfaction with ‘Cost’, followed by ‘Comfort’ and ‘Follow-up.’ For different types of orthoses, the highest mean total score was 4.30 (SD = 0.57) for pressure garments, and the lowest mean total score was 3.40 (SD = 0.66) for knee-ankle-orthoses. The scores of the items rated by the participants with pressure garments were all above 4, except the ‘Comfort’ item.

**Table 3:** Means (SD) of satisfaction scores of T-QUEST for each orthosis and for all orthoses.

	FO	AFO	KAFO	HKAFO	SB	PG	All orthoses
	(n = 30)	(n = 57)	(n = 85)	(n = 36)	(n = 38)	(n = 34)	(n = 280)
Device (mean)							3.74 (0.64)
Dimension	3.63 (0.62)	3.81 (0.69)	3.65 (0.80)	3.75 (0.69)	3.76 (0.75)	4.35 (0.69)	3.79 (0.75)
Weight	3.67 (0.66)	3.86 (0.74)	3.24 (0.95)	3.25 (1.03)	3.71 (0.80)	4.41 (0.66)	3.62 (0.92)
Adjustment	3.70 (0.79)	3.77 (0.89)	3.42 (0.88)	3.72 (0.91)	3.82 (0.65)	4.38 (0.65)	3.73 (0.87)
Safety	3.77 (0.77)	4.05 (0.74)	3.69 (0.76)	4.00 (0.54)	3.89 (0.69)	4.50 (0.56)	3.94 (0.74)
Durability	3.73 (0.74)	3.82 (0.85)	3.58 (0.79)	3.89 (0.62)	3.95 (0.77)	4.18 (0.83)	3.81 (0.80)
Ease of use	3.90 (0.55)	3.75 (0.99)	3.53 (0.92)	3.50 (0.94)	3.87 (0.53)	4.24 (0.89)	3.74 (0.88)
Comfort	3.77 (0.63)	3.67 (1.01)	3.19 (1.02)	3.17 (0.97)	3.37 (0.91)	3.91 (1.11)	3.46 (1.00)
Effectiveness	3.77 (0.57)	3.75 (0.93)	3.69 (0.79)	3.75 (0.84)	3.84 (0.82)	4.35 (0.65)	3.82 (0.81)
Service (mean)							3.56 (0.76)
Service delivery	3.70 (0.70)	3.67 (1.04)	3.40 (0.86)	3.58 (0.81)	3.82 (0.80)	4.24 (0.70)	3.67 (0.88)
Repairs & services	3.70 (0.75)	3.77 (0.82)	3.41 (0.94)	3.86 (0.80)	3.58 (0.89)	4.50 (0.56)	3.73 (0.89)
Professional services	3.77 (0.63)	3.95 (0.74)	3.52 (0.93)	3.72 (0.88)	3.89 (0.73)	4.53 (0.66)	3.83 (0.85)
Follow-up	3.50 (0.90)	3.58 (0.74)	3.20 (1.02)	3.47 (0.97)	3.16 (0.86)	4.26 (0.83)	3.47 (0.98)
Cost	3.20 (0.89)	3.61 (1.02)	2.66 (1.07)	2.47 (1.00)	2.97 (0.92)	4.09 (0.75)	3.10 (1.11)
Total	3.68 (0.48)	3.77 (0.60)	3.40 (0.66)	3.55 (0.51)	3.66 (0.51)	4.30 (0.57)	3.67 (0.64)

FO: Foot Orthosis, AFO: Ankle Foot Orthosis, KAFO: Knee Ankle Foot Orthosis, SB: Spinal Brace, PG: Pressure Garment.

The results indicated a significant difference between the mean subscores of T-QUEST for all six types of orthotic devices and for service (p = 0.001). The mean service subscore was lower compared to the device subscore. The three items of T-QUEST that were ranked as the most important were ‘Comfort’ (53.2%), ‘Safety’ (42.9%), and ‘Cost’ (32.1%). The three least important items identified by the users were ‘Service delivery’ (5.4%), ‘Adjustment’ (7.9%), and ‘Follow-up’ (8.6%).

### Comparison of satisfaction scores

There was no significant difference in device, service and total satisfaction scores between males and females or among age groups, and educational levels. Only one significant difference emerged in service score (p = 0.002) among participants living in different areas. Post-hoc tests indicated that the service score in participants who living in the eastern area and offshore islands was significantly lower than those in participants who live in the other areas (all p<0.05). The other pair groups did not differ significantly from one another. A significant difference was found in device, service and total satisfaction scores among severity of disability (all p = 0.015), types of orthoses (all p = 0.001) and duration of usage (all p = 0.001). Post-hoc tests revealed that the satisfaction scores in participants with mild disability was significantly higher than that in participants with moderate or severe disability (all p<0.05), while the latter two groups did not differ significantly from one another. The satisfaction scores in participants who using a pressure garment were significantly higher than that in participants who using the other types of orthoses (all p<0.05). There was not a significant difference of satisfaction scores between all pairs of groups using the other types of orthoses. Post-hoc analysis showed that all pairs were significantly different (all p<0.05) except for the pairs (3–5y, ≥5y) regarding the duration of usage. The results showed that the longer the duration of usage, the lower the satisfaction scores. The participants who were using the orthoses for less than one year were the most satisfied with the orthotic device and service.

### Reasons for being dissatisfied

In contrast to previous studies that examined users' satisfaction with one specific orthosis, this study investigated users' satisfaction with six types of orthoses. Since all these orthoses were funded through the government welfare allowance, this study was commissioned by a government program for investigating the user satisfaction under the subsidy scheme. Furthermore, by including six types of orthoses it was possible to compare the user satisfaction with different types of orthoses. It was speculated that the reasons for dissatisfaction stated in each item might concern one specific type of orthosis. However, the reasons for dissatisfaction were applicable to all types of orthoses after the data was reviewed with scrutiny. For each item, participants who assigned score of less than 4 to different types of orthoses gave the same reasons for dissatisfaction. The percentages of participants who indicated dissatisfied (score of less than 4) and provided comments for each item in each orthosis were shown in Table 4.

**Table 4:** The percentages of participants who indicated dissatisfied and provided comments for each item in each orthosis.

Items	FO	AFO	KAFO	HKAFO	SB	PG	Total number of comments
Dimension	40.0% (4/10)	46.7% (7/15)	51.9%(14/27)	40.0% (4/10)	37.5% (3/8)	0% (0/2)	32
Weight	63.6% (5/9)	57.1% (8/14)	74.5% (35/47)	75.0% (12/16)	41.7%(5/12)	0% (0/1)	65
Adjustment	37.5% (3/8)	26.7% (4/15)	27.8% (10/36)	44.4% (4/9)	25.0% (2/8)	100% (1/1)	24
Safety	75.0% (6/8)	62.5% (5/8)	48.1% (13/27)	40.0% (2/5)	25.0% (2/8)	0% (0/2)	28
Durability	71.4% (5/7)	75.0%(6/8)	71.4% (25/35)	71.4% (5/7)	55.6%(5/9)	57.1% (4/7)	50
Ease of use	60.0% (3/5)	70.6% (12/17)	54.5% (18/33)	63.6% (7/11)	37.5% (3/8)	50.0% (3/6)	45
Comfort	50.0% (4/8)	50.0% (10/20)	54.2% (26/48)	44.4% (8/18)	58.8% (10/17)	63.6% (7/11)	65
Effectiveness	25.0% (2/8)	20.0% (3/15)	31.0% (9/29)	25.0% (4/10)	33.3% (3/9)	33.3% (1/3)	22
Service delivery	50.0% (4/8)	44.4% (8/18)	54.5% (24/44)	57.1% (8/14)	33.3% (3/9)	33.3% (1/3)	48
Repairs & services	37.5% (3/8)	23.5% (4/17)	32.5% (13/40)	50.0% (4/8)	28.6% (4/14)	0% (0/1)	28
Professional services	28.6% (2/7)	26.7% (4/15)	28.6% (10/35)	25.0% (3/12)	22.2% (2/9)	0% (0/1)	21
Follow-up	50.0% (5/10)	50.0% (12/24)	51.1% (24/47)	57.1% (8/14)	50.0% (11/22)	66.7%(4/6)	64
Cost	70.6% (12/17)	57.9% (11/19)	75.0% (48/64)	82.8% (24/29)	50.0% (11/22)	66.7% (4/6)	110

FO: Foot Orthosis, AFO: Ankle Foot Orthosis, KAFO: Knee Ankle Foot Orthosis, SB: Spinal Brace, PG: Pressure Garment.

Note. Denominators in the parentheses: number of participants who indicated dissatisfied; numerators in the parentheses: number of participants who provided comments.

The most common reasons that the users were not quite satisfied or very satisfied included the following: 1) Dimension: Most participants (n = 32) commented that the orthoses did not fit properly and were either too large or too small; 2) Weight: Most participants (n = 65) commented that the orthoses were too heavy; 3) Adjustment: Fewer number of participants (n = 24) commented about the problems related to adjustment of the orthoses; 4) Safety: Numerous participants (n = 28) commented on safety issue. Some of the comments stated that the orthoses were lacking security and stability because the screws were too loose or the soles of the shoes were very slippery, 5) Durability: Most participants (n = 50) commented about the poor durability of the accessories of the orthoses, such as padded foam, velcro, insoles, or leather, 6) Ease of use: Some participants (n = 45) expressed problems with the use of the orthoses. They commented that it was difficult for them to put the orthoses on or take off, 7) Comfort: Most participants (n = 65) stated that they were uncomfortable when wearing the orthoses for a long period because they felt hot and the orthoses had poor air permeability, 8) Effectiveness: Some respondents (n = 22) stated that their problems had not been resolved or the effects of orthoses were different from what had been expected; 9) Service delivery: Most participants (n = 48) complained about the service processes or procedures, stating that they were long and complicated, 10) Repairs and services: Some participants (n = 28) commented on this issue, including that the warranty period was too short, there were no maintenance services, the repairs were time-consuming, there were no in-house services, and service attitudes were poor, 11) Professional services: Participants (n = 21) also commented about professional services. The participants expressed that the quality of professional services was not high enough, as no professional assessment was provided, the professionals were not full participation and lack of proper information (e.g., wearing methods, maintenance) provided, 12) Follow-up: Most of the participants (n = 64) stated that there were no follow-up services, 13) Cost: All participants (n = 110) commented that the cost of the orthoses was too high.

### Opinions and additional suggestions

Additional suggestions were made about the orthotic devices, including the need to use new, advanced materials to increase durability (fatigue-resistance), strength, permeability and comfort, as well as allow for a greater choice of styles for footwear and consider the cosmetic appearance of orthoses. Suggestions were also made about orthotic service, including ensuring the availability and accessibility of the service needed, improving the efficiency of repair or maintenance service, providing orthotic evaluations and training by an appropriately trained professional, and providing complete and current information concerning the orthoses (e.g., manufacturer, specification, and price).

Some additional suggestions were made about subsidy policy; including the maximum subsidy funding should be increased. Participants further suggested that the items of auxiliary appliances covered in the benefit categories should be increased, the application process should be simplified, and the minimum lifespan of appliances (i.e., time period for reapplication) should be reduced.

## Discussion

In this study, we examined users' satisfaction with orthotic devices and service using T-QUEST. Although various versions of QUEST were not explicitly designed to measure user satisfaction with orthoses, and they may not embrace all important aspects of satisfaction specific to orthotic users [26], T-QUEST was the only culture-specific measurement of user satisfaction that possessed good psychometric properties when used with Taiwanese people [16]. Moreover, the participants were asked to provide additional comments and suggestions revealing the important aspects of their orthoses (e.g., cosmetic appearance of device) in addition to those revealed in the measurement.

The results showed that about two-thirds of the participants were quite satisfied or very satisfied with their devices and service. The mean satisfaction score was 3.74 for the devices and 3.56 for service, and these values were lower compared to those obtained from satisfaction surveys conducted in western countries, where the average satisfaction scores were 4 or more [27,28]. However, the satisfaction scores were similar to those obtained from the other studies using T-QUEST or the Chinese version of QUEST (C-QUEST). Since most Chinese people express their opinions conservatively, they would choose the middle (near score 3) rather than the extreme answers [12,16]. Certainly, there is still room for improvement in orthotic devices and service in Taiwan. Specifically, more effort should be put on improving orthotic service since the satisfaction with services was lower than satisfaction with the orthotic devices.

The item ‘Safety’ was rated high on both importance and satisfaction; thus, this strength should be highlighted. In contrast, the item ‘Follow-up’ was rated low on importance and low on satisfaction. Although ‘Follow-up’ was not rated as a very important item, it still worthy to invest efforts to improve follow-up service. The two items ‘Comfort’ and ‘Cost’ were both rated high on importance and low on satisfaction. This means that there was a large gap between the perceived level of importance and the perceived level of satisfaction and that these areas deserve immediate attention. The items that were identified as low on satisfaction and high on importance should be given the highest priority.

The costs of the appliances were not fully covered by the welfare allowance; the government reimbursed only a portion of the expense. The orthotic users must pay the remaining, non-reimbursed portion. Aside from the non-reimbursed expenses, the users must also pay the maintenance and repair fees for their orthoses. Furthermore, a considerable number of the participants (45%) were unemployed or low-income employees. Therefore, ‘Cost’ was a major concern for the users of the orthoses. Most participants also suggested increasing the maximum subsidy for the devices and service. Another low satisfaction item, ‘Comfort,’ was also the only item that received a score of less than 4 for the pressure-garment users. A previous study showed that discomfort appeared to promote low compliance with pressure garment use [29], and satisfaction with comfort appeared to be an important factor for device usage [30]. Therefore, the orthotist or therapist must assess whether the orthosis causes the user any discomfort or pain when prescribing an orthosis.

‘Cost’, ‘Comfort’, ‘Control’, and ‘Cosmesis’ are called the four C's, and they are four most important concepts that should be considered when defining an “ideal” orthosis for a patient [31]. The term ‘Control’ means controlling function and achieving the desired goal, which is equivalent to ‘Effectiveness’ in our measurement. ‘Cosmesis’ was not included in T-QUEST, but some participants suggested that cosmetic appearance of orthoses should be considered. Previous studies have also demonstrated that persons often want their orthoses to be cosmetically appealing, convenient to use with their usual clothing, and not too noticeable [32,33].

Our results revealed no significant gender, age, and educational level differences in satisfaction scores. The results were consistent with the findings of Geertzen's study in which it was demonstrated that consumer satisfaction with prosthetic and orthotic facilities did not differ by age and gender [17]. However, the results of Hall and Dornan's study showed that older and less educated patients were more satisfied with their medical care, while gender did not correlate with satisfaction [34]. Inconsistent results in the literature indicated that sociodemographic characteristics were only minor predictors of satisfaction [6]. Exceptionally, the participants from eastern area and offshore islands were the least satisfied with the orthotic service. In Taiwan, the eastern area and offshore islands are considered remote areas. The resources for providing orthotic service in remote areas are less than in urban area. Therefore, the government has a responsibility for improving access to orthotic service in eastern area and offshore ilands in Taiwan.

In our study, the participants with mild disability were most satisfied with the device and service. The possible reason is that the participants with mild disability were likely to have less impairment, and it appears that it is easier to reduce mild disability caused by impairment rather than moderate or severe disability. Hall and colleagues reviewed the literature and found that patients with better health status tend to be more satisfied with their medical care, but their study did not confirm the causal factors in this relationship [35]. The pressure-garment users also obtained the highest satisfaction score. They rated all items except for ‘Comfort’, a score of 4 or more. Perhaps some items of T-QUEST were less relevant to pressure garments, such as weight, adjustments, and safety, leading to fewer problems in those items. Previous study also demonstrated that the factors that affect burn patients' satisfaction with pressure therapy included fees, knowledge, effectiveness, mobility, and comfort [36]. Some factors (e.g., knowledge, mobility) presented in that study were different from the satisfaction items in T-QUEST. The participants who used their orthoses for less than one year had the greatest satisfaction scores. This finding could be possibly attributed to the new users were more easily to perceive the effectiveness of the device when compaired to no orthosis and the problems in some items (e.g., durability and repair) were less likely to occure in a short period of time. On the contrary, the longer the duration of usage, the problemns were likely to occure.

Some participants expected the service provider to provide orthotic evaluation and training by an appropriately trained professional and allow them to access complete and current information concerning the orthoses. The Ministry of Health and Welfare in Taiwan established the Center for Assistive Technology Resources and Popularization to integrate resources and promote service of assistive technology. In addition, a website (Resource Portal of Assistive Technology) was created to provide information about subsidies and welfare, legislation and regulations, product specifications and vendors, and assistive technology service. The website enables service users to obtain updated and versatile information, and receive suitable services effectively [37].

The reasons for being dissatisfied as well as the opinions and suggestions proposed by the participants may direct orthotic manufacturers and professionals to fabricate state-of-the-art orthoses and deliver high-quality service and guide the government in their policy decisions. The satisfaction of users measured by T-QUEST provided additional culture-specific information concerning the cost. Most users felt that the orthosis was too expensive and hoped that the maximal subsidy funding could be increased. Moreover, the participants living in the eastern area and offshore islands were the least satisfied with orthotic service. These results may give a reference to related studies conducted in different countries with similar economic situation and subsidy policies.

One limitation of the current study was that the small, non-probability convenience sample may limit the generalizability of this study. Another limitation was that we did not ask participants how many hours per day they wore their orthoses, since most participants wore their orthoses every day. The wearing time per day may affect the satisfaction.

## Conclusion

In conclusion, this study demonstrated that the overall user satisfaction with orthotic devices and service were median high. About two-thirds of users were quite satisfied or very satisfied with their orthotic devices and service. The participants with mild disability, wearing the pressure garment and using the orthosis for less than one year, were the most satisfied. There is a need for improved orthotic devices and service, especially for improved comfort and subsidy funding. The satisfaction of orthoses users can be improved by using advanced materials that would increase permeability and comfort, ensuring availability and accessibility of service, increasing the maximum subsidy for orthoses, and the like. The results of this study and the suggestions made by the participants provide useful information for clinicians to better understand the needs of the users of orthoses and for policymakers to consider in future policy making.
